# Dual inhibition of sodium–glucose linked cotransporters 1 and 2 exacerbates cardiac dysfunction following experimental myocardial infarction

**DOI:** 10.1186/s12933-018-0741-9

**Published:** 2018-07-07

**Authors:** Kim A. Connelly, Yanling Zhang, Jean-François Desjardins, Kerri Thai, Richard E. Gilbert

**Affiliations:** Keenan Research Centre, Li Ka Shing Knowledge Institute, St. Michael’s Hospital, 61 Queen Street East, Toronto, ON M5C 2T2 Canada

**Keywords:** Sodium–glucose linked cotransporter, Myocardial infarction, Systole, Diastole

## Abstract

**Background:**

Inhibiting both type 1 and 2 sodium–glucose linked cotransporter (SGLT1/2) offers the potential to not only increase glucosuria beyond that seen with selective SGLT2 inhibition alone but to reduce glucose absorption from the gut and to thereby also stimulate glucagon-like peptide 1 secretion. However, beyond the kidney and gut, SGLT1 is expressed in a range of other organs particularly the heart where it potentially assists GLUT-mediated glucose transport. Since cardiac myocytes become more reliant on glucose as a fuel source in the setting of stress, the present study sought to compare the effects of dual SGLT1/2 inhibition with selective SGLT2 inhibition in the normal and diseased heart.

**Methods:**

Fischer F344 rats underwent ligation of the left anterior descending coronary artery or sham ligation before being randomized to receive the dual SGLT1/2 inhibitor, T-1095, the selective SGLT2 inhibitor, dapagliflozin or vehicle. In addition to measuring laboratory parameters, animals also underwent echocardiography and cardiac catheterization to assess systolic and diastolic function in detail.

**Results:**

When compared with rats that had received either vehicle or dapagliflozin, T-1095 exacerbated cardiac dysfunction in the post myocardial infarction setting. In addition to higher lung weights, T-1095 treated rats had evidence of worsened systolic function with lower ejection fractions and reduction in the rate of left ventricle pressure rise in early systole (dP/dt_max_). Diastolic function was also worse in animals that had received T-1095 with prolongation of the time constant for isovolumic-pressure decline (Tau) and an increase in the end-diastolic pressure volume relationship, indices of the active, energy-dependent and passive phases of cardiac relaxation.

**Conclusions:**

The exacerbation of post myocardial infarction cardiac dysfunction with T-1095 in the experimental setting suggests the need for caution with the use of dual SGLT1/2 inhibitors in humans.

## Background

Sodium–glucose linked cotransporter-2 inhibitors (SGLT2i) increase urinary glucose excretion thereby off-loading energy, lowering plasma glucose and body weight as well as inducing a modest diuresis that reduces extracellular fluid volume and lowers blood pressure. In addition, two recent cardiovascular outcome studies with these agents demonstrated reductions in heart failure hospitalisation suggesting that this class of drug exerts cardioprotective effects beyond glucose and blood pressure lowering [1, 2].

In addition to SGLT2, another glucose transporter, SGLT1, cotransports sodium and glucose in the distal segment of the proximal tubule with higher affinity and lower capacity than its more proximal counterpart. Unlike SGLT2, whose expression is confined to the kidney, SGLT1 is also abundantly present in the gut where it accounts for much of glucose (and galactose) absorption by enterocytes. Accordingly, combined SGLT1/2 inhibition offers the potential to not only increase glucosuria beyond that seen with SGLT2 inhibition alone but to reduce glucose absorption allowing the monosaccharide to stimulate release of glucagon-like peptide-1 (GLP-1) in the ileum. As such, dual SGLT1/2 inhibition presents an appealing strategy for glucose lowering in diabetes with drugs that do so currently in development [3].

Beyond its expression in the kidney and small intestine, however, SGLT1 is also present in various other organs in humans including the lung, liver, pancreatic alpha cells, skeletal muscle and particularly in the heart where its abundance exceeds that of the kidney [4–6]. Similar high expression levels have recently also been reported in the hearts of rats and mice [7]. With its constant and high energy requirements and little storage capacity, the heart requires a constant supply of energy-generating substrates. This is especially important during ischaemia when the ability to generate more ATP per O_2_ molecule consumed and to also generate ATP anaerobically by glycolysis render glucose preferable to fatty acids as a substrate for energy production [8]. Although the facilitated glucose transporters GLUT1 and GLUT4 were previously thought to account entirely for glucose transport in the heart, more recent studies attest to the additional contribution of SGLT1 [9–12].

Given their potential to enter the clinical arena, we sought to examine the effects of dual SGLT1/2 inhibition in experimental heart disease using the rat coronary artery ligation model that develops ischemia, infarction and heart failure. Accordingly, the primary objective of the study was to assess changes in cardiac function and secondarily changes in structure. To avoid the requirement for longterm parenteral administration with the poorly absorbed and rapidly metabolized phlorizin, we administered the readily absorbed SGLT1/2 dual inhibitor prodrug, T-1095, that is quickly converted to the active moiety, T-1095A following its entry into the systemic circulation [13, 14].

## Methods

### Animals

Ninety-nine 7-week old male rats Fischer F344 rats (Charles River Laboratories, Wilmington, MA, USA) were randomized to myocardial infarction or sham groups in a ratio of 3:1 using the left anterior descending (LAD) coronary artery of myocardial infarction that provides a robust model of adverse cardiac remodeling akin to its human counterpart [15]. Animals were then further randomized in a ratio of 1:1:1 to receive either vehicle, the dual SGLT1/2 inhibitor, T-1095 (150 mg/kg/day, Sun-Shine Chemical Technology Co., Ltd, Shanghai, China) dosed as previously described [16], or the selective SGLT2 inhibitor, dapagliflozin 0.5 mg/kg b.i.d (gift of Astra Zeneca, Gothenburg, Sweden) [17] by gavage. Doses of T-1095 (150 mg/kg/day or 0.1 wt/wt admixed in chow) and dapagliflozin (1 mg/kg/day) were based on previously published literature according to their ability to achieve an approximate 50% reduction in plasma glucose when administered to diabetic rats [18, 19].

Myocardial infarction was induced 1 week later by ligation of the LAD coronary artery with sham animals undergoing thoracotomy and incision of the pericardial sac, but not LAD ligation [20]. Two days later, rats underwent echocardiography, and were randomized on confirmation of MI and demonstration of similar MI size based upon fractional shortening and wall motion score index [21] and followed for a further 7 weeks before being terminated. All procedures were performed in the research vivarium under anesthesia using 2.5% isoflurane supplemented with 100% O_2_ as previously undertaken and shown to effectively reduce consciousness without impairing cardiac function [22]. Urine was obtained for measurement of glucose concentration just prior to termination by cervical dislocation under isoflurane anesthesia. Because of the comparatively short duration of the study in comparison with the half-life of erythrocytes, fructosamine rather than hemoglobin A_1c_ was used provide an integrated index of glycemia. Serum fructosamine concentrations was, accordingly, determined by colorimetric assay using a commercial diagnostic assay (Roche Diagnostics, Canada) analyzed on the Olympus AU400 automated chemistry analyzer (Beckman Coulter, Canada).

Animals underwent echocardiography and cardiac catheterization as described below. Following these procedures, rats were terminated and their hearts and lungs removed for weighing and other assessments, as also described below. Tibial length was measured to provide a morphometric index for cardiac hypertrophy and lung weight [23].

Sample size estimates were based on similar studies previously reported by our group using this animal model [24]. Of the cohort of 99 rats, five did not show evidence of wall motion abnormalities indicative of infarction when echocardiography was performed 2 days after LAD ligation. A further 17 (8 from the vehicle group and 9 from the dapagliflozin group died during surgery or during the immediate postoperative recovery period following myocardial infarction. Two post-MI animals were withdrawn from the study when it became evident that there were insufficient quantities of T-1095 for them to complete. As such, the final number of animals that completed the study as per protocol were 22, 13 and 15 among the MI + vehicle, MI + dapagliflozin and MI + T-1095 groups, respectively and 12, 7 and 6 among the sham + vehicle, sham + dapagliflozin and sham + T-1095 groups, respectively.

All animals were housed 2/cage at the St. Michael’s Hospital Animal Research Vivarium in a temperature-controlled (22 °C) room with a 12-h light/dark cycle and ad libitum access to commercial standard rat chow. Enrichment, proper animal handling and anesthesia procedure were used to minimize the pain and distress of the animals during the study. All animal studies were approved by the St. Michael’s Hospital Animal Care Committee in accordance with the Guide for the Care and Use of Laboratory Animals (NIH Publication No. 85-23, revised 1996).

### Echocardiography

Transthoracic echocardiography was performed, as previously described [25], under light anaesthesia (1% isoflurane supplemented with 100% O_2_), at 2 days and 8 weeks post MI, prior to sacrifice. Images were acquired using a high-frequency ultrasound system (Vevo 2100, MS-250 transducer, Visualsonics, Toronto, ON). Two dimensional long-axis images of the LV in parasternal long- and short-axis views with M-mode measurements at mid-papillary muscle level and linear dimensions were analyzed offline (Vevo 2100 software v. 1.8) using the standard leading edge-to-leading edge technique by a single investigator, masked to treatment.

MI size was estimated 2 days after LAD ligation by measuring the percentage of endocardial circumferential extent of LV akinesis at end-diastole using 2D short-axis images of the LV at mid-papillary muscle level. Corrected LV mass was calculated using the Devereux and Reichek “cube” formula that includes a 20% correction for overestimation of LV mass based on data derived from previously reported validation studies [26, 27]. Fractional shortening (FS%) was calculated according to the formula: FS% = (LVIDd − LVIDs)/LVIDd × 100, where LVIDd and LVIDs are end-diastolic diameter and end-systolic diameter respectively, as previously described [28]. Three consecutive cardiac cycles were averaged for all analyses.

### Cardiac catheterization

Cardiac catheterization was performed as previously published [25]. Briefly, rats were anaesthetized with 2.5% isoflurane, intubated using a 14 gauge catheter and ventilated using a pressure controlled ventilator (TOPO ventilator, Kent Scientific, Torrington, CT). Adequacy of anaesthesia was assessed by lack of response to surgical manipulation and loss of muscular tone. Rats were placed in the supine position on a water circulating heating pad and a 1.4 F pressure–volume (PV) catheter (SPR-838; Millar Instruments, Inc., Houston, TX) was inserted into the right carotid and advanced into the left ventricle and PV loops were generated. All pressure–volume (PV) loops were obtained with the ventilator turned off for 5–10 s and the animal apnoeic.

Data were acquired and recorded with a MPVS ultra^®^ data acquisition system (Millar Instruments) and LabChart Pro software (CHART 8.1 ADInstruments Inc., Colorado Springs, CO) under steady-state and following inferior vena cava occlusion (preload reduction). Conductance signals acquired with the Millar catheter were calibrated with estimated LV volumes derived echocardiographically using a two-point calibration method, matching LV maximal and minimal conductance signals and end-diastolic and end-systolic volumes (EDV and ESV) measured in long-axis view for each individual rat, respectively. Using the pressure conductance data, a range of functional parameters were then calculated.

### Histopathology

To determine the extent of extracellular matrix deposition, sections were stained with antibodies to types I and III collagen (Southern Biotech, Birmingham, AL). The abundance of matrix within the non-infarct zone was then quantified as previously described [29]. To isolate the non-infarct zone from the infarct and the peri-infarct zone, the infarct and a 2 mm zone on either side of it were excluded from analysis. The proportional area staining brown within the remaining myocardium composed the non-infarct zone was then assessed using computer-assisted image analysis (Aperio ImageScope, Leica Biosystems Inc., Concord, Ontario, Canada), as previously described [30]. The whole non-infarct zone was used for quantification of ECM in order to prevent possible bias from using selected fields. For sham animals, the ECM content of the entire LV was quantitated by the same method, as described above.

The extent of cardiac myocyte hypertrophy was determined on haematoxylin and eosin stained sections, as previously reported [31]. In brief, stained sections were scanned with the Aperio Ultra-Resolution Digital Scanning System (Aperio Technologies Inc., Vista, CA), and the images were analyzed with The NDP.view2 viewer software (Hamamatsu Photonics, Hamamatsu City, Shizuoka Pref., Japan). Cardiac myocytes with elliptical nuclei in transverse section were selected. Diameter measurements were taken membrane to membrane across the narrowest point that crosses the nucleus. The average diameter of 30–50 myocytes per animal was measured based on the method previously reported [32].

### Western blot

The abundance of SGLT 1 and 2 were assessed by immunoblot, as previously described [33] using specific isoform antibodies (Santa Cruz Biotechnology, Dallas, TX). In brief, small pieces of heart tissue were rapidly collected from the non-infarct zone, snap frozen in liquid nitrogen and stored at − 80 °C until use. Lysates were prepared by homogenizing frozen heart tissue in ice-cold lysis buffer (50 mM Tris, pH 7.4, 150 mM NaCl, 1 mM EDTA, 1 mM EGTA, 0.5% Triton X-100 containing 1 mM Na_3_VO_4_, 50 mM NaF, 25 mM β-glycerophosphate, 10 mM Na pyrophosphate, 10 µg/ml aprotinin, 10 µg/ml leupeptin, 1 mM PMSF, 1 mM DTT). After 30 min incubation on ice, lysates were centrifuged to remove cell debris. Protein concentration was determined using Bradford reagent (Bio-rad, Hercules, CA) and standardized with known amounts of BSA. Western blot analysis was performed by resolving 50 µg of total protein on a 10% SDS-polyacrylamide gel by denaturing discontinuous gel electrophoresis according to the Laemmli method, and transferring to PVDF membranes (Roche, Mannheim, Germany). After incubating in blocking solution (5% nonfat dry milk, Tris buffered saline (pH 7.5), 0.1% Tween 20), membranes were immunoblotted with primary antibodies at 1:1000 dilution overnight at 4 °C. Membranes were then washed three times (5 min each) with TBST and blots were incubated with HRP-conjugated secondary antibodies (Dako) for 1 h at room temperature. The membranes were washed three times, and proteins were detected by the ECL system (Roche). Rat kidney served as positive control for SGLT2. Signals were digitized then analyzed with ImageJ software (NIH, Bethesda, MD). Results were calculated as the mean of at least three experiments relative to ß-actin immunolabelling. Kidney homogenates served as positive controls for both SGLT1 and − 2.

### Gene expression

The abundance of atrial natriuretic peptide (ANP) was assessed by measuring its mRNA by quantitative real-time PCR in left ventricular tissue stored at − 80 °C as previously described [34]. In brief, SYBR green-based measurement of gene expression were performed on QuantStudio 7 Flex Real-Time PCR System (Applied Biosystems, Foster City, CA) according to the manufacturer’s instructions using the predesigned sequence-specific primers for ANP from Integrated DNA Technologies (Coralville, IA). Data were analyzed using Applied Biosystems Comparative CT method.

### Statistics

Data are expressed as mean ± SEM unless otherwise specified. Between group differences were analyzed by one way ANOVA with Fisher’s Protected Least Significant Difference test post hoc. All statistics were performed using GraphPad Prism 6 for Mac OS X (GraphPad Software Inc., San Diego, CA). A p value of < 0.05 was regarded as statistically significant.

## Results

### Morphometric characteristics

No differences in baseline in body weight among the six groups were evident prior to randomization. When compared with rats that had been treated with dapagliflozin, those randomized to receive T-1095 had lower body weights in the post-MI setting (Table 1). Left ventricular weight, indexed to tibial length, increased following myocardial infarction with lower values noted in rats that had received dapagliflozin but lower still in those randomized to T-1095. Lung weight indexed to tibial length was increased in all animals that had undergone coronary artery ligation but to a greater extent in rats that had received T-1095 compared with dapagliflozin-treated animals.

**Table 1:** Animal characteristics and laboratory parameters

	sham + vehicle	sham + dapa	sham + T-1095	MI + vehicle	MI + dapa	MI + T-1095
*N*	12	7	6	22	13	15
BW (g)	305.3 ± 3.8	281.3 ± 3.3*	248.3 ± 8.1*	299.9 ± 3.1	282.8 ± 5.0*^†^	263.5 ± 4.2*^†^
LV W/TL (g/mm)	14.8 ± 0.1	13.4 ± 0.1*	12.2 ± 0.3*	15.9 ± 0.2*	15.0 ± 0.2^†^	14.1 ± 0.4^†^
Lung W/TL (g/mm)	27.4 ± 0.5	26.8 ± 0.3	24.7 ± 0.4	43.7 ± 3.9*	40.3 ± 4.4*	54.2 ± 5.0*^†^
UG (mmol/l)	0.34 ± 0.07	316.0 ± 34.3*	256.2 ± 66.6*	0.21 ± 0.03	235.0 ± 10.3*^†^	205.2 ± 21.6*^†^
Fructosamine (μM)	141.8 ± 3.3	126.2 ± 2.4*	129.2 ± 2.8*	121.5 ± 3.3*	122.5 ± 2.0*	130.8 ± 4.4*^†^
ANP: RPL13a mRNA (AU)	1.09 ± 0.23	1.39 ± 0.44	1.52 ± 0.23	13.02 ± 2.33*	7.47 ± 1.15*^†^	9.61 ± 1.07*

*BW* body weight, *SBP* systolic blood pressure, *LVW* left ventricle weight, *TL* tibia length, *Lung W* lung weight, *BG* blood glucose, *UG* urinary glucose, *ANP* atrial natriuretic peptide, *RPL13a* Ribosomal Protein L13a, *AU* arbitrary units, *dapa* dapagliflozin

* p < 0.05 vs. sham + vehicle group; ^†^ p < 0.05 vs. MI + vehicle group

### Laboratory and molecular parameters

**Fig. 1:**
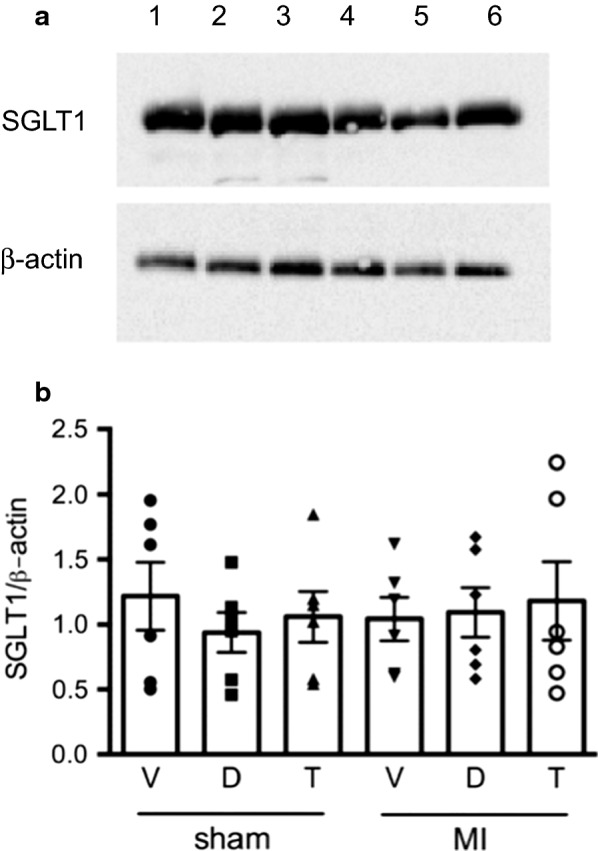
SGLT1 expression. Western blot of cardiac tissue showing similar abundance of SGLT1 relative to ß-actin in all groups. N = 6/group

Fructosamine concentrations were lower in both dapagliflozin and T-1095 when compared with vehicle-treated control animals. In rats that had undergone LAD ligation, however, fructosamine concentrations were lower than in vehicle-treated control animals though less so among rats that had received T-1095 (Table 1). No glucosuria was detected in untreated animals but was readily apparent in animals that received either T-1095 or dapagliflozin (Table 1). SGLT1 was easily detected by immunoblot of cardiac tissue but was unaffected by either myocardial infarction or treatment group assignment (Fig. 1). SGLT2 on the other hand, while abundantly expressed in the kidney could not be detected in cardiac tissue (data not shown).

The expression of ANP mRNA was assessed as a marker of extracellular fluid volume and cardiac wall stress. Its abundance was increased in the hearts of all animals that had undergone experimental myocardial infarction but less so in those that had received dapagliflozin when compared with either vehicle or T-1095 treated rats (Table 1).

### Echocardiography

**Fig. 2:**
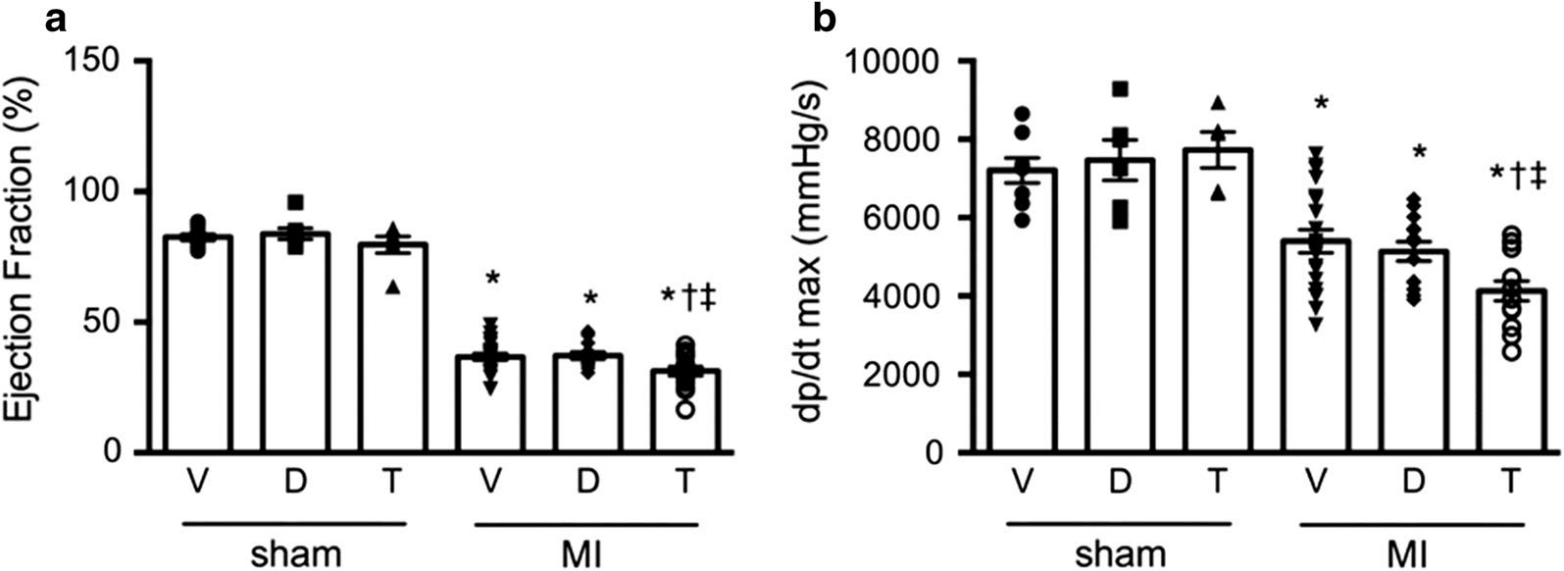
Systolic function. Systolic function measured as ejection faction by echocardiography (**a**) and rate of left ventricle pressure rise in early systole (dP/dt_max_) by conductance catheterization (**b**). Animal numbers are provided in Tables 2 and 3. * p < 0.05 vs. sham + vehicle, ^†^ p < 0.05 vs. MI + vehicle, ^‡^ p < 0.05 vs. sham + dapagliflozin

Ejection fraction and fractional shortening were reduced in all animals that undergone experimental myocardial infarction when compared with sham surgery animals (Fig. 2, Table 2). Ejection fraction was, however, reduced to an even greater extent in those rats that had received T-1095 than those treated with either vehicle or dapagliflozin. Similar changes were also noted in left ventricular mass that while increased in all groups that had undergone LAD ligation, this was less so in those that had received T-1095 when compared with either vehicle- or dapagliflozin-treated animals.

**Table 2:** Echocardiographic parameters

	sham + vehicle	sham + dapa	sham + T-1095	MI + vehicle	MI + dapa	MI + T-1095
*N*	12	7	6	20	12	14
MI size (%)	–	–	–	44 ± 2	38 ± 2	40 ± 2
FS (%)	53 ± 1	55 ± 3	50 ± 3	19 ± 1*	19 ± 1*	16 ± 1*
EF (%)	83 ± 1	84 ± 2	80 ± 3	37 ± 1*	37 ± 1*	31 ± 2*^†‡^
LV mass corr. (mg)	684 ± 26	608 ± 35*	578 ± 41	827 ± 36*	800 ± 41*	759 ± 39
LVIDd (mm)	6.73 ± 0.08	6.62 ± 0.18*	6.50 ± 0.07*	9.17 ± 0.15*	9.21 ± 0.18*	9.05 ± 0.11*
LVIDs (mm)	3.17 ± 0.09	3.01 ± 0.25	3.25 ± 0.18	7.47 ± 0.17*	7.48 ± 0.20*	7.64 ± 0.11*
Volume s (μl)	63 ± 4	52 ± 6	56 ± 10	359 ± 28*	371 ± 32*	402 ± 18*
Volume d (μl)	289 ± 15	272 ± 19	223 ± 13	574 ± 25*	555 ± 31*	556 ± 19*

*EF* ejection fraction, *FS* fractional shortening, *LV mass corr* left ventricle mass corrected, *LVPW* left ventricular posterior wall, *LVIDd* left ventricle internal diameter in diastole, *LVIDs* left ventricle internal dimension in systole

* p < 0.05 vs. sham + vehicle group; ^†^ p < 0.05 vs. MI + vehicle group; ^‡^ p < 0.05 vs. MI + dapagliflozin group

### Conductance catheterization

**Fig. 3:**
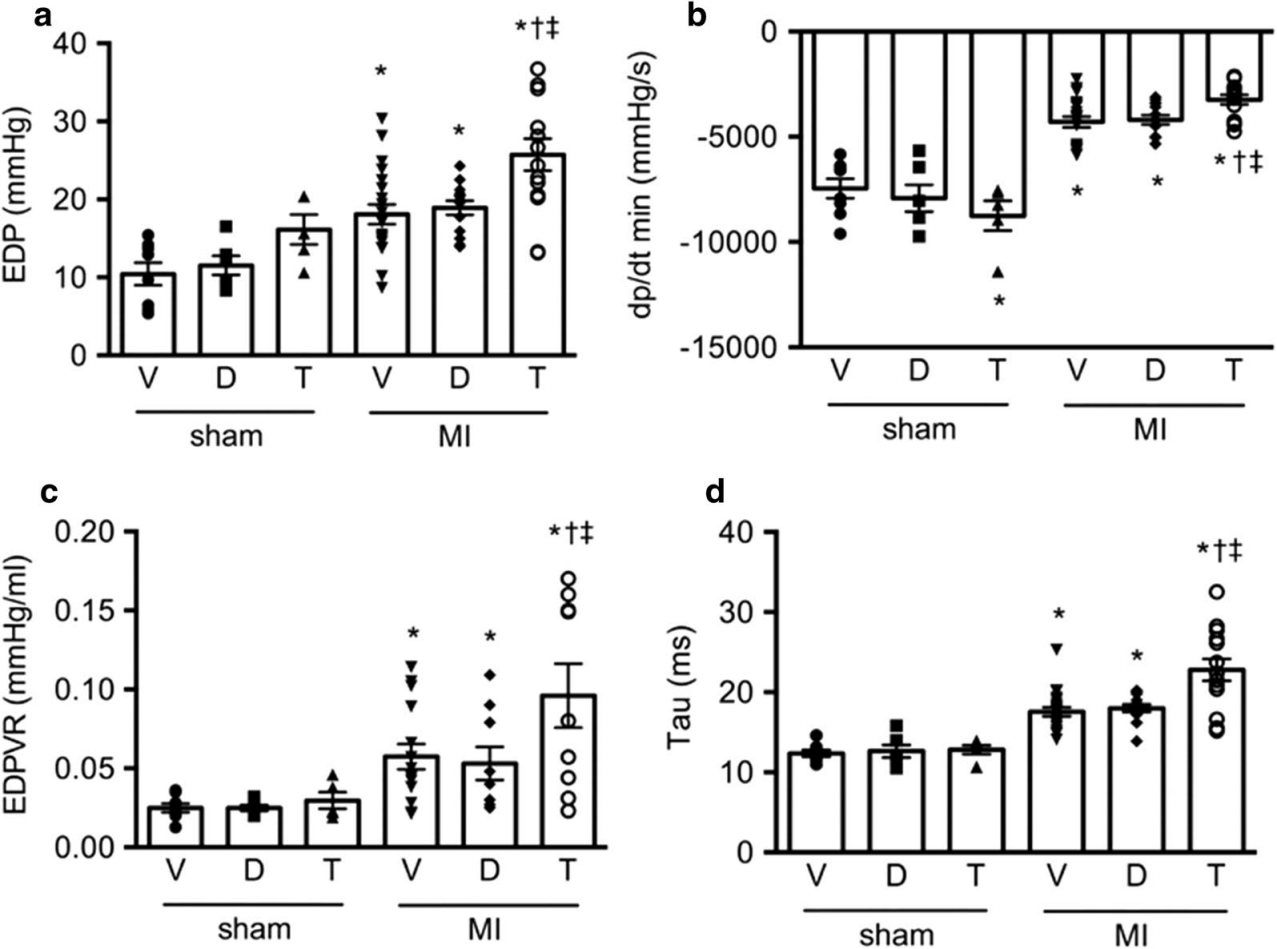
Diastolic function. Diastolic function measured as left ventricular end-diastolic pressure (EDP, **a**), the early decrease in LV pressure during isovolumic relaxation (dP/dt_min_, **b**), end-diastolic pressure volume relationship (EDPVR, **c**) and the diastolic time constant, Tau (**d**), all measured by conductance cardiac catheterization. Animal numbers are provided in Table 3. * p < 0.05 vs. sham + vehicle, ^†^ p < 0.05 vs. MI + vehicle, ^‡^ p < 0.05 vs. MI + dapagliflozin

Global left ventricular contractility, as measured by the rate of left ventricle pressure rise in early systole (dP/dt_max_), was reduced in the post-MI setting but to a greater extent in those that had received T-1095 when compared with either vehicle or dapagliflozin-treated animals (Fig. 2, Table 3). The rapid, early decrease in LV pressure during the period of isovolumic relaxation (dP/dt_min_) that reflects diastolic function was also reduced in rats that had undergone LAD ligation (Fig. 3). The extent of the reduction differed according to treatment assignment with those animals randomized to receive T-1095 experiencing the greatest reduction. This same pattern was also evident in other indices of diastolic function including both Tau, that assesses the early energy-dependent phase of relaxation as well as the end-diastolic pressure volume relationship (EDPVR) that provides an index of passive LV compliance (Fig. 3, Table 3). Systolic blood pressure was similar in all groups with the exception of post-MI rats receiving T-1095 where it was slightly lower.

**Table 3:** Conductance catheterization

	sham + vehicle	sham + dapa	sham + T-1095	MI + vehicle	MI + dapa	MI + T-1095
*N*	5	6	4	20	13	13
EDP (mmHg)	11 ± 2	12 ± 1	16 ± 2*	18 ± 1*	19 ± 1*^‡^	26 ± 2*^†^
Pes (mmHg)	117 ± 11	127 ± 7	127 ± 15	107 ± 5	105 ± 5	95 ± 5*
HR (bpm)	296 ± 12	288 ± 12	299 ± 11	270 ± 7*	258 ± 6*	260 ± 9*
dP/dt_max_ (mmHg/s)	7031 ± 426	7471 ± 514	7815 ± 504	5443 ± 305*	5141 ± 249*	4201 ± 264*^†‡^
dP/dt_min_ (mmHg/s)	− 7190 ± 631	− 7922 ± 637	− 8927 ± 906*	− 4318 ± 260*	− 4192 ± 224*	− 3290 ± 246*^†‡^
EDPVR (mmHg/ml)	0.024 ± 0.004	0.025 ± 0.002	0.03 ± 0.01	0.06 ± 0.01	0.05 ± 0.01	0.10 ± 0.02*^†‡^
Tau (ms)	12.5 ± 0.6	12.6 ± 0.8	12.5 ± 0.7	17.5 ± 0.6*	18.0 ± 0.5*	23.2 ± 1.6*^†‡^

*EDP* end-diastolic pressure, *Pes* end-systolic pressure, *HR* heart rate, *EDPVR* end-diastolic pressure volume relationship, *ESPVR* end-systolic pressure volume relationship

* p < 0.05 vs. sham + vehicle group; ^†^ p < 0.05 vs. MI + vehicle group; ^‡^ p < 0.05 vs. MI + dapagliflozin group

### Histopathology

**Fig. 4:**
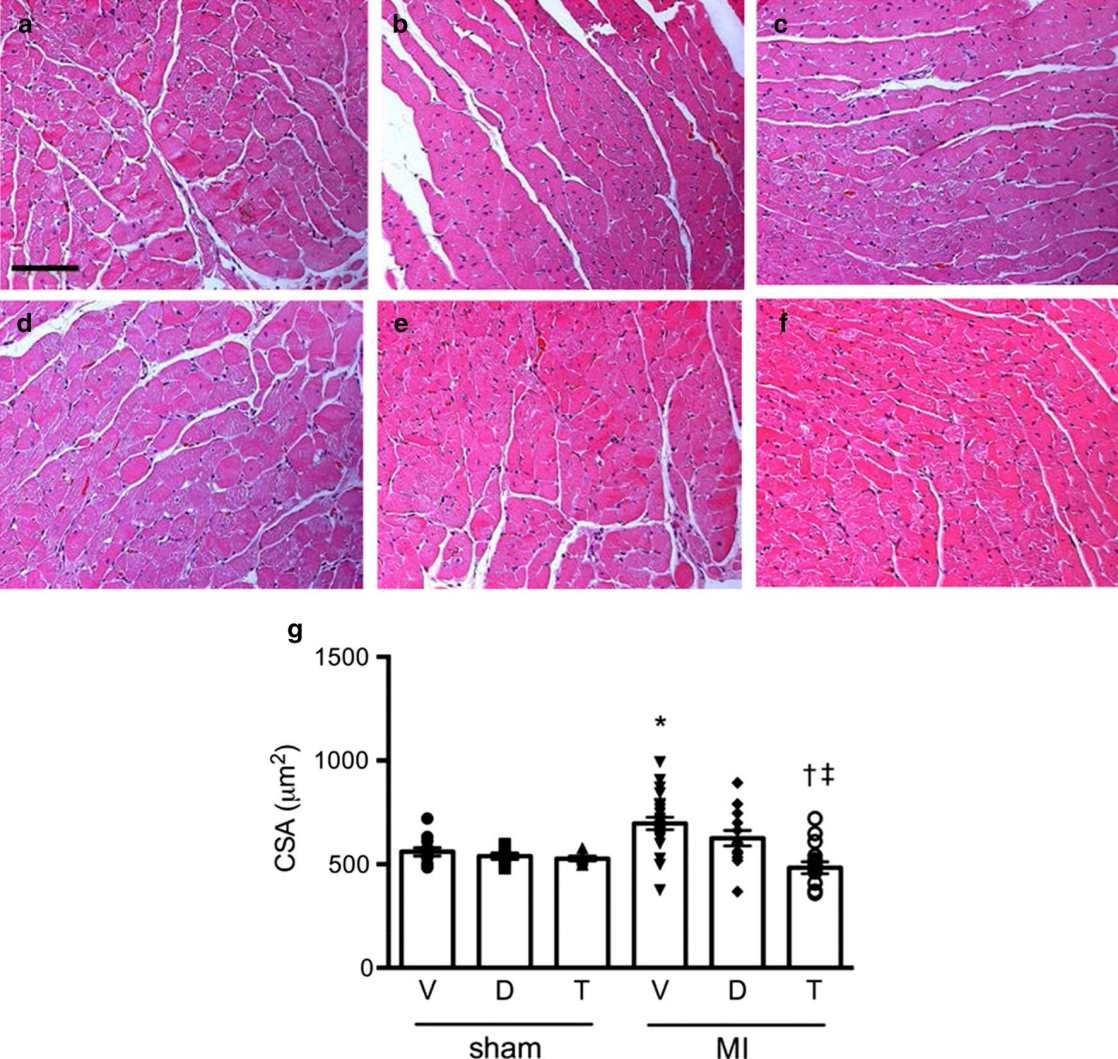
Myocyte size. Hematoxylin and eosin-stained cardiac tissue from rats receiving vehicle (**a**, **d**), dapagliflozin (**b**, **e**) or T-1095 (**c**, **f**) in sham (**a**–**c**) and post-MI (**d**–**f**) settings along with quantitative analysis of myocyte size (**g**). Animal numbers are provided in Table 1. Scale bar: 100 microns. * p < 0.05 vs. sham + vehicle, ^†^ p < 0.05 vs. MI + vehicle, ^‡^ p < 0.05 vs. MI + dapagliflozin

**Fig. 5:**
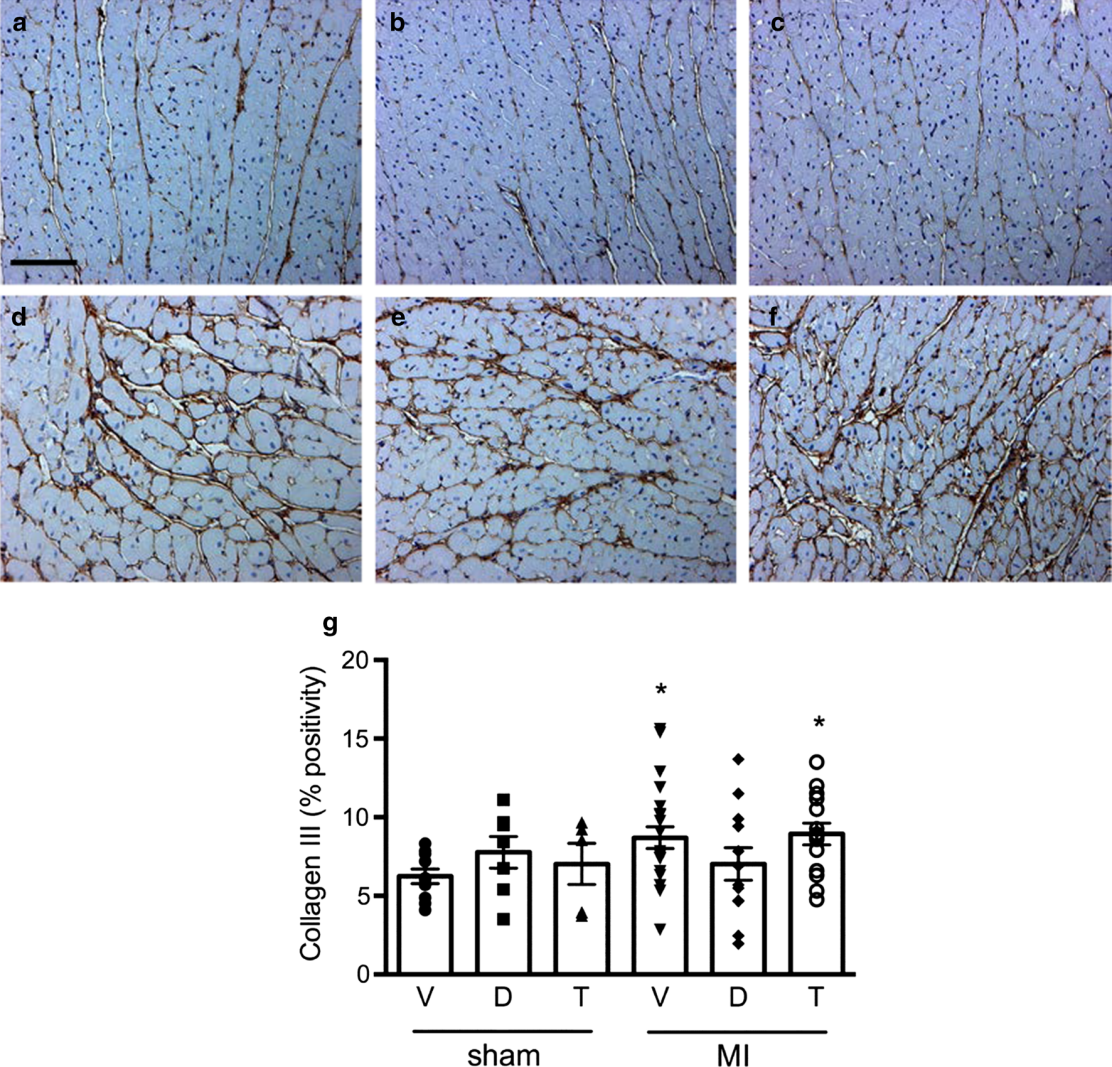
Collagen deposition. Immunolabelling of collagen III in hearts from rats receiving vehicle (**a**, **d**), dapagliflozin (**b**, **e**) or T-1095 (**c**, **f**) in sham (**a**–**c**) and post-MI (**d**–**f**) settings along with quantitative analysis of its abundance (**g**). Scale bar: 100 microns. * p < 0.05 vs. sham + vehicle

**Fig. 6:**
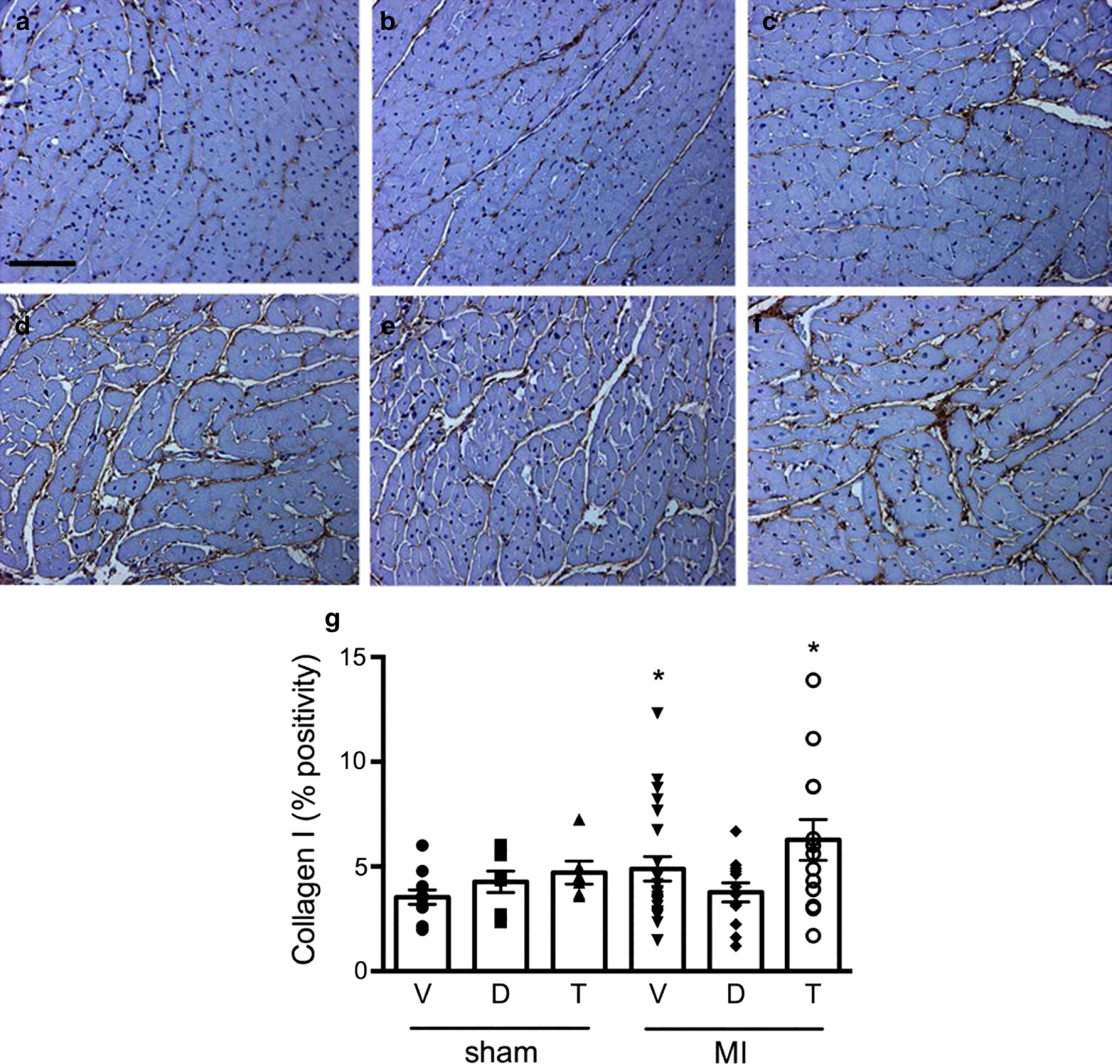
Collagen deposition. Immunolabelling of collagen I in hearts from rats receiving vehicle (**a**, **d**), dapagliflozin (**b**, **d**) or T-1095 (**c**, **e**) in sham (**a**–**c**) and post-MI (**d**–**f**) settings along with quantitative analysis of its abundance (**g**). Scale bar: 100 microns. * p < 0.05 vs. sham + vehicle

Myocyte dimensions differed according to treatment assignment. Animals that received T-1095 did not show evidence of hypertrophy in the post-MI setting, contrasting those in rats randomized to vehicle or dapagliflozin (Fig. 4). The extent of fibrosis in the left ventricle in the area remote from the site of infarction also differed according to group assignment. Increased type III collagen was evident in the interstitium of those rats that had received either vehicle or T-1095 but not in those that had been treated with dapagliflozin (Figs. 5, 6). A different pattern was observed for type I collagen where increased deposition was only found in animals that had received T-1095 following experimental myocardial infarction (Figs. 5, 6).

## Discussion

Unlike SGLT2 whose expression is almost exclusively confined to the kidney, SGLT1 is abundantly expressed in the heart. Blocking the SGLT2 transporter alone with dapagliflozin had no effect on cardiac function in either the control setting or after myocardial infarction. Dual blockade of SGLT 1 and 2 with T-1095, however, while not affecting function under physiological circumstances led to an exacerbation of impairment in both systole and diastole in the post myocardial infarction setting.

Ligation of the left anterior descending coronary artery leads to substantial infarction of the left ventricle, providing a well-established model of progressive heart failure in rodents [15]. As expected, all animals in the current study that had undergone ligation displayed evidence of systolic dysfunction. Notably, the key features of left ventricular function such as ejection fraction and dP/dt_max_ were both worse in the group that had received the SGLT1/2 inhibitor, T-1095 when compared with those treated with either vehicle or dapagliflozin. Similarly, diastolic function was also abnormal 4 weeks following LAD ligation but worse still among those rats that had received T-1095. Moreover, abnormalities in both the early, active, energy-dependent phase of diastole as indicated by prolongation of the diastolic time constant, Tau and the later, more passive phase of diastole as reflected by an increase in the end-diastolic pressure volume relationship (EDPVR) were also more severely affected in T-1095-treated animals.

Following ischaemic necrosis of the myocardium the heart undergoes adaptive (in addition to adverse) remodelling in an attempt to compensate for the loss of contractile tissue. In this regard, an increase in left ventricular wall thickness as a consequence of myocyte hypertrophy reduces wall stress, as dictated by the Laplace law [35]. Notably, the requirement for anabolic growth in addition to maintaining ATP generation for cardiac contractility necessitates an increase in glycolytic flux [36]. In this setting, the role of SGLT1-mediated glucose entry into cardiac myocytes that can be inhibited by agents such as T-1095 becomes increasingly important [11, 37, 38]. Indeed, inhibition of cardiac hypertrophy in the setting of increased wall stress, as demonstrated in the present study would be predicted to be detrimental [39] as demonstrated by the cardiac dysfunction and reduced survival when hypertrophy is inhibited in experimental animals subjected to cardiac stress [40–44]. In contrast to the aforementioned primary inhibition of myocyte hypertrophy, ACE inhibitors and β-blockers reduce preload and afterload so that the need for hypertrophy is reduced and reverse remodeling may occur.

Beyond the effects on myocyte hypertrophy, we also observed increased collagen deposition in animals that had received T-1095. As described above, T-1095 mediated inhibition of SGLT1 leads to a diminution in glucose entry that reduces ATP generation, consistent with the reduction in systolic function (EF, dp/dt_max_) and the impairment in the active phase of diastolic relaxation (Tau) reported in the present study. In addition to impaired function, however, the resultant energy depletion would also provide a profibrotic stimulus [45] that would lead to the increase in interstitial collagen.

Phlorizin, the prototypical SGLT1/2 inhibitor is poorly absorbed from the gut so that in addition to a low plasma concentration following oral dosing, its high luminal concentration induces diarrhoea by inhibiting enterocyte SGLT1. To overcome these limitations, we took advantage of T-1095, a phlorizin derivative prodrug that is readily absorbed from small intestine prior to being converted to its active form, T-1095A, in the systemic circulation [13]. Similar to phlorizin that is a near-equipotent inhibitor of SGLT1 and 2, T-1095A has only a fourfold selectivity for SGLT2 over SGLT1 [14]. In contrast, the three widely-marketed SGLT2 inhibitors, canagliflozin, dapagliflozin and empagliflozin that have SGLT2:SGLT1 selectivity ratios in the order of > 250, > 1200 and > 2500, respectively [46] and while canagliflozin may inhibit gut SGLT1 in clinically used doses [47], its plasma concentration would not be expected to reach levels high enough to inhibit cardiac SGLT1. Beyond canagliflozin, however, drugs with even less selectivity are in development, seeking to capitalise on the theoretical additional glucose lowering that may be achieved by inhibiting SGLT1 in the distal segments of the proximal tubule as well as SGLT2 in the more proximal segments [48]. Notably, two drugs designed to inhibit SGLT1 have already entered clinical trials. Sotagloflozin (LX4211) with an IC_50_ ratio of 20:1 in favour of SGLT2 has completed phase 3 while others such as GSK-1614235 (Glaxo Smith-Kline, PA), designed to preferentially inhibit SGLT1 with an IC_50_ that is 300-fold less for SGLT1 versus SGLT2, has recently undergone phase 1 clinical testing [49].

The present study has several limitations. First and foremost, while a commonly used model, the abrupt induction of ischemic necrosis in a rodent heart has only superficial resemblance to the myocardial infarction in humans that occurs in the setting of longstanding atherosclerotic disease, collateral development and in whom a vast array of cardioprotective measures are now employed. Notably, clinical studies of SGLT2 inhibitors have not shown evidence of benefit in being able to reduce the risk of myocardial infarction. As such, the absence of significant improvement in cardiac parameters with dapagliflozin should not be interpreted as an indication that this compound does not prevent hospitalization for heart failure in the clinical setting especially considering the positive findings of its phase 2/3 program [50], post-marketing studies [51] and the effects of other members of the SGLT2 inhibitory class [52] along with supporting basic and translational research studies [53–55]. Secondly, the compound used to inhibit SGLT1/2, T-1095, is not one that is in current use and while the drug was used as a pharmacological probe, extrapolations to other chemical entities may not be warranted. In particular, the combined SGLT1/2 inhibitor, sotagliflozin, as noted previously, has a 20:1 selectivity in favour of SGLT2, contrasting the 4:1 ratio for T-1095.

Although T-1095A’s ability to inhibit SGLT1-mediated glucose uptake has been well-documented [13, 14, 16], we did not directly assess glucose transport into the myocardium in the current study. Several other groups have explored the effects of SGLT1 inhibition in heart in cells, atrial strips and using the ex vivo Langendorff technique with T-1095 and phlorizin [10, 11, 38, 56]. These studies demonstrated the importance of SGLT1-mediated glucose transport in the stress setting whereby its inhibition exacerbates ischemia–reperfusion injury [38, 56]. The novelty and potential clinical relevance of the current study, however, centers on it being the first to examine the effects of SGLT1 inhibition following cardiac injury in the in vivo setting. Notably, given SGLT-mediated effects on preload and afterload [57], we used conductance catheterization that unlike echocardiography provides load-independent measurements of cardiac function in intact animals [25]. While the plasma concentration of T-1095A was not measured in the current study, its effects on SGLT-1 mediated glucose transport can be inferred from the IC_50_ for rat SGLT1 of < 1 µM [14] and the plasma concentration of ~ 10 µM that, according to published pharmacokinetic for the compound, would have been attained with the doses used [58]. Finally, although a direct effect of T-1095 on cardiac SGLT1 provides a cogent explanation for the observed effects, it is conceivable that altered glucose transport in other cells that express SGLT1 might have contributed, including the cardiac capillary endothelium [4].

## Conclusions

The exacerbation of post myocardial infarction cardiac dysfunction with T-1095 in the experimental setting suggests the need for caution with the use of dual SGLT1/2 inhibitors in humans, reinforcing the prudent approach taken by regulatory authorities in specifically studying patients with cardiovascular disease before bringing a new glucose-lowering drug to market.

## Abbreviations

ANP: atrial natriuretic peptide
BG: blood glucose
BP: blood pressure
BW: body weight
dP/dt_max_: rate of left ventricle (LV) pressure during isovolumic contraction
dP/dt_min_: (− dP/dt) pressure fall during isovolumic relaxation
EDPVR: end-diastolic pressure volume relationship
EF: ejection fraction
FS: fractional shortening
LAD: left anterior descending coronary artery
MI: myocardial infarction
SGLT: sodium–glucose linked cotransporter
TL: tibial length
